# Marine Omega-3 (N-3) Fatty Acids for Cardiovascular Health: An Update for 2020

**DOI:** 10.3390/ijms21041362

**Published:** 2020-02-18

**Authors:** Jacqueline K. Innes, Philip C. Calder

**Affiliations:** 1 School of Human Development and Health, Faculty of Medicine, University of Southampton, Southampton SO16 6YD, UK; innesjackie@gmail.com; 2 National Institute for Health Research Southampton Biomedical Research Centre, University Hospital Southampton NHS Foundation Trust and University of Southampton, Southampton SO16 6YD, UK

**Keywords:** eicosapentaenoic acid, docosahexaenoic acid, omega-3 polyunsaturated fatty acids, cardiovascular disease, coronary heart disease

## Abstract

The omega-3 (n-3) fatty acids, eicosapentaenoic acid (EPA) and docosahexaenoic acid (DHA), are found in seafood (especially fatty fish), supplements and concentrated pharmaceutical preparations. Long-term prospective cohort studies consistently demonstrate an association between higher intakes of fish, fatty fish and marine n-3 fatty acids (EPA + DHA) or higher levels of EPA and DHA in the body and lower risk of developing cardiovascular disease (CVD), especially coronary heart disease (CHD) and myocardial infarction (MI), and cardiovascular mortality in the general population. This cardioprotective effect of EPA and DHA is most likely due to the beneficial modulation of a number of known risk factors for CVD, such as blood lipids, blood pressure, heart rate and heart rate variability, platelet aggregation, endothelial function, and inflammation. Evidence for primary prevention of CVD through randomised controlled trials (RCTs) is relatively weak. In high-risk patients, especially in the secondary prevention setting (e.g., post-MI), a number of large RCTs support the use of EPA + DHA (or EPA alone) as confirmed through a recent meta-analysis. This review presents some of the key studies that have investigated EPA and DHA in the primary and secondary prevention of CVD, describes potential mechanisms for their cardioprotective effect, and evaluates the more recently published RCTs in the context of existing scientific literature.

## 1. Marine Omega-3 Fatty Acids: Sources and Intakes

Omega-3 (n-3) fatty acids are a family of polyunsaturated fatty acids. They are characterised by, and named according to, the presence of the closest double bond to the methyl end of the hydrocarbon (acyl) chain being on carbon number three, if the methyl carbon is counted as number one. The most functionally important n-3 fatty acids appear to be eicosapentaenoic acid (EPA; 20:5n-3) and docosahexaenoic acid (DHA; 22:6n-3) [1]; however, roles for docosapentaenoic acid (22:5n-3) have also emerged now [2]. The best dietary source of EPA and DHA (and also docosapentaenoic acid) is seafood, especially fatty fish (also called ‘oily fish’). The blubber and tissues of sea mammals, such as whales and seals, also contain EPA and DHA in significant amounts. Various supplements, including fish oils, cod liver oil, krill oil and some algal oils, contain EPA and DHA. Finally, concentrated pharmaceutical-grade preparations of EPA and DHA, or EPA alone, are available. Typical values for the EPA and DHA content of selected fish, n-3 fatty acid supplements and pharmaceutical preparations are shown in Table 1. EPA and DHA are often referred to as marine n-3 fatty acids because of their association with seafood.

**Figure 1:**
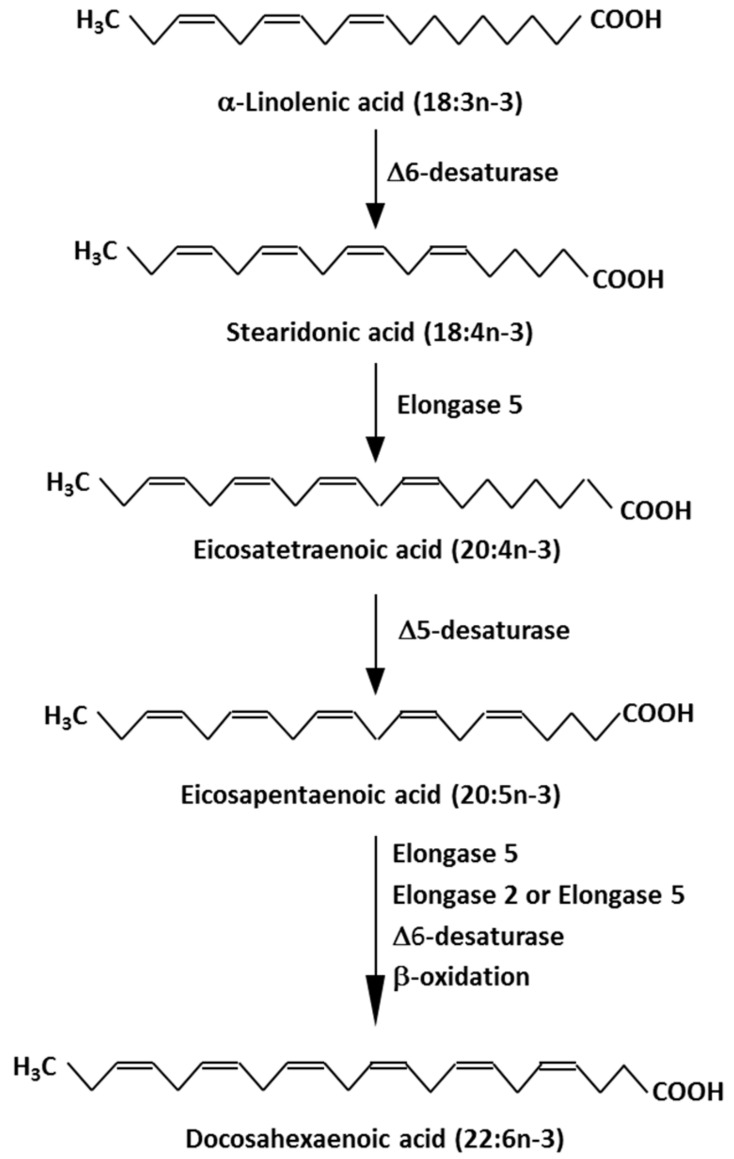
Metabolic pathway of conversion of the plant essential n-3 fatty acid, α-linolenic acid (18:3n-3), to eicosapentaenoic acid (20:5n-3) and docosahexaenoic acid (22:6n-3).

The various n-3 fatty acids are related metabolically to one another, and the pathway of conversion of plant-derived n-3 fatty acids (e.g., α-linolenic acid (ALA; 18:3n-3)) to EPA and then to DHA is shown in Figure 1. Studies in humans have identified that there is a fairly low rate of conversion of ALA along this pathway, especially all the way to DHA [3,4]. It is now recognised that this conversion is influenced by several factors, including the stage of life course, age, sex, various hormones, genetics and diseases [4].

The intake of EPA and DHA from diet is strongly influenced by fish consumption because fish, in general, and fatty fish, in particular, are the richest dietary source of these fatty acids. The intake of fish and fatty fish is high in some countries, such as Japan, but it is low in many Western countries, including the USA and the United Kingdom. As a result, the intake of EPA + DHA among adults varies among different populations and is low in most Western countries; it is generally considered that in non-fatty fish eaters, the intake of EPA + DHA is <0.2 g/day [5,6]. This is lower than the recommended intake for the general population [7,8,9]. Nevertheless, despite these low intakes, it is evident that the recommendations (typically 0.2–0.5 g/day depending upon the authority making the recommendation) can be met by including fatty fish in the diet on a regular basis or, if that is not possible, by using supplements that contain EPA and DHA (Table 1).

## 2. Strong Evidence for a Protective Effect of EPA and DHA Towards Cardiovascular Disease Emerges from Ecological, Case Control and Cohort Studies

The potential for EPA and DHA to have a role in reducing the risk of cardiovascular disease (CVD) was first identified by studies in the Greenland Inuit, where the low rate of mortality from myocardial infarction (MI) and ischaemic heart disease [10,11] was linked to the very high dietary intake of EPA and DHA [12]. These observations were replicated in other native Arctic populations [13] and the Japanese population [14]. Subsequently, substantial evidence accumulated from epidemiological and case-control studies in Western populations indicating that consumption of fish, fatty fish, or EPA and DHA is associated with reduced risk of mortality from CVD, especially coronary heart disease (CHD) (reviewed in [15]). For example, in the Nurse’s Health Study, there was an inverse dose-dependent association of risk for developing CHD, having a non-fatal MI or dying from CHD across quintiles of intake of EPA + DHA [16]. The risk for all three outcomes was about 50% in the group with the highest intake compared with the group with the lowest intake of EPA+DHA. The intake of EPA and DHA is highly correlated with their concentrations in blood lipids and red blood cells [17]. A number of studies have associated the concentrations of EPA + DHA (often expressed as a proportion of total fatty acids) in blood plasma or serum, plasma lipid fractions, whole blood, red blood cells and adipose tissue with lower cardiovascular morbidity and mortality (reviewed in [15]). For example, in the Physician’s Health Study, there was an inverse dose-dependent association of risk for sudden death across quartiles of whole blood EPA + DHA, with an 80% lower risk in those with the highest whole blood EPA+DHA concentration compared to those with the lowest whole blood EPA + DHA concentration [18]. More recently, the largest prospective cohort study conducted to date included ~420,000 participants from the National Institutes of Health AARP Diet and Health Study with a 16-year follow-up and reported a significant inverse association between fish and EPA + DHA intake and various mortality outcomes [19]. Comparing the highest with lowest quintiles of fish intake, both men and women had 10% lower CVD mortality. EPA + DHA intake was associated with 15% and 18% lower CVD mortality in men and women, respectively, across extreme quintiles.

Cohort studies associating the intake of fish or marine n-3 fatty acids with cardiovascular or coronary outcomes have been subject to a number of meta-analyses. These include a 2012 aggregation of seven prospective cohort studies, including 176,441 participants, which investigated the association between dietary fish, EPA+DHA intake or plasma EPA + DHA concentrations and heart failure [20]. The investigators found a 15% risk reduction of heart failure associated with the highest versus lowest fish intake and a 14% lower risk of heart failure for those with the highest intake compared to those with the lowest dietary intake or plasma concentrations of EPA + DHA. A comprehensive meta-analysis, published in 2014, investigated the association between dietary intakes or blood levels of different classes of fatty acids (including n-3 fatty acids) and combined coronary disease outcomes [21]. The aggregation of data from 16 studies involving over 422,000 individuals showed a risk reduction of 13% for those in the top tertile of dietary EPA + DHA intake compared with those in the lower tertile of intake. Furthermore, the aggregation of data from 13 studies involving over 20,000 individuals showed risk reductions of 22%, 21% and 25% for those in the top tertile of circulating EPA, DHA and EPA + DHA, respectively, compared with those in the lower tertile. Alexander et al. [22] brought together data from prospective cohort studies examining the association of dietary EPA and DHA with risk of various coronary outcomes. The aggregation of data from 17 studies showed an 18% risk reduction for any CHD event for those with higher dietary intake of EPA + DHA compared to those with lower intake. There were also significant reductions of 23%, 19% and 47% in the risk for fatal coronary events, coronary death and sudden cardiac death, respectively.

The association between EPA or DHA concentration in a body compartment, such as plasma, serum, red blood cells or adipose tissue, and risk of future CHD in adults who were healthy at study entry was investigated by pooling data from 19 studies involving over 45,000 individuals [23]. EPA and DHA were each independently associated with a lower risk of fatal CHD, with a 10% lower risk for each one standard deviation increase in content [23]. The omega-3 index is the red blood cell content of EPA+DHA expressed as a proportion of total fatty acids [24]. Omega-3 index is a marker of both long-term dietary intake of these fatty acids and their tissue levels and is suggested to be a marker of CHD risk [24]. Harris et al. [25] used data from 10 cohort studies and identified a 15% reduction in risk of fatal CHD for each one standard deviation increase in omega-3 index.

## 3. Mechanisms by which EPA and DHA Reduce the Risk of Cardiovascular Disease

Prospective cohort studies have the advantage of a very long follow-up time to observe health outcomes in what starts as a generally healthy study population, something which is typically not possible in randomised control trials (RCTs). There are well-recognised limitations of such cohort studies, including the lack of ability to show causation. Despite this significant limitation, the considerable number of large prospective cohort studies conducted to date that have consistently shown an inverse association between dietary, blood or tissue EPA and DHA and incidence of mortality from CVD provide important evidence for the key role of marine n-3 fatty acids in the prevention of CVD. As such, there has been much interest in the mechanisms by which n-3 fatty acids, specifically EPA and DHA, achieve their cardioprotective action, with much attention being focused on the potential modulation of key cardiovascular risk factors. These risk factors include high blood pressure, high serum triglycerides, low high-density lipoprotein (HDL)-cholesterol, elevated post-prandial lipaemia, endothelial dysfunction, cardiac arrhythmia, heart rate and heart rate variability and a tendency towards thrombosis and inflammation. Large numbers of studies, including many RCTs, in humans have investigated the effect of the combination of EPA and DHA on these risk factors and many of these studies have been included in a number of meta-analyses performed in recent years (Table 2). These meta-analyses demonstrate that EPA and DHA lower triglycerides [26], lower the blood pressure (both systolic and diastolic) [26,27], reduce the heart rate and increase heart rate variability [26,28,29,30] and reduce platelet aggregation [31], whilst appearing to increase both low density lipoprotein (LDL)- and HDL-cholesterol [26]. Regarding vascular endothelial function, EPA and DHA have been demonstrated to improve flow-mediated dilatation [32,33] and arterial compliance [34]. Concerning the effect of EPA and DHA on inflammation, several meta-analyses have reported that they lower blood concentrations of the acute phase protein, C-reactive protein (CRP), and the pro-inflammatory cytokines, tumour necrosis factor (TNF)-α and interleukin (IL)-6 [26,35], although the effect may be dependent on the health status of the individual. Furthermore, EPA and DHA have been reported to decrease the plasma or serum concentrations of pro-inflammatory eicosanoids like thromboxane B_2_ and leukotriene B_4_ [36].

Whilst the majority of the scientific evidence base to-date has focused on the administration of EPA and DHA in combination (as occurs naturally in fish and most supplements), there has been much interest in the potential for EPA and DHA to have independent roles in cardiovascular risk reduction. A recent systematic review of the scientific literature concluded that EPA and DHA appear to have differential effects on a number of cardiometabolic outcomes [37]. For example, regarding modulation of blood lipids, whilst both EPA and DHA lowered blood triglycerides, there was evidence for a slightly larger triglyceride-lowering effect for DHA [38,39]. Whilst neither EPA nor DHA affected total cholesterol concentrations to a significant degree, there was an independent effect on other blood lipid parameters, with EPA lowering the HDL3-cholesterol subfraction and DHA increasing the more cardioprotective HDL2-cholesterol [40,41]. DHA also increased LDL-cholesterol more than EPA, an effect observed more in men than in women, and increased LDL particle size, an effect which was not observed with EPA [39,40,41]. From the more limited trial data, DHA appears to be more effective than EPA at lowering blood pressure and heart rate in normotensive individuals, whilst neither EPA nor DHA had any effect in hypertensive diabetic patients [40,41,42,43]. DHA also appeared to increase vasodilatory effects and reduce constrictor effects in the vasculature [44]. Both EPA and DHA were equally effective at increasing systemic arterial compliance [45]. In terms of platelet function, only EPA decreased platelet count and volume [46], whilst only DHA decreased collagen-stimulated platelet aggregation and platelet-derived thromboxane B_2_ [47]. Interestingly, neither EPA nor DHA had any effect on fibrinolytic function [47]. Furthermore, from the limited comparative studies available, DHA seemed to be more effective than EPA at lowering a wide range of pro-inflammatory biomarkers in subjects with subclinical inflammation [39,48]. Both EPA and DHA, however, were effective at reducing biomarkers of oxidative stress (F2 isoprostanes) [49,50,51]. Thus, whilst there are relatively few trials that have been conducted to date that directly compare EPA and DHA, the limited data suggest that EPA and DHA have different effects with regard to cardiovascular risk factors. More research, however, is necessary to be more certain about this.

## 4. RCTs of Primary Prevention of Cardiovascular Disease with Marine n-3 Fatty Acids

When compared to the large number of observational studies investigating the association between marine n-3 fatty acid exposure and cardiovascular outcomes that have been conducted to date (Section 2), only a few RCTs of sufficient size or duration have investigated the cardioprotective effects of marine n-3 fatty acids in generally healthy populations. The open-label, Japan EPA Lipid Intervention Study (JELIS) directly investigated the use of 1.8 g/d EPA (as an ethyl ester) plus statin versus statin alone in 18,645 hypercholesterolaemic participants [52]. A number of the participants were on existing cardiovascular medication (in addition to statin) and the study included hypercholesterolemic, but otherwise, healthy subjects as well as those with pre-existing CHD, with all patients being followed up for ~5 years. The primary outcome was any major coronary event, including sudden cardiac death, fatal and non-fatal MI and other non-fatal events, including unstable angina pectoris, angioplasty, stenting, and coronary artery bypass grafting. The addition of EPA to statin had no effect over statin alone on the primary outcome in the primary prevention arm of the trial. Two large primary prevention RCTs were published in late 2018 [53,54]. The ASCEND (A Study of Cardiovascular Events iN Diabetes) trial randomised 15,480 people with diabetics and no evidence of CVD to receive either marine n-3 fatty acids (840 mg/d EPA + DHA) or olive oil placebo [53]. The primary outcome was the first serious vascular event and after a mean follow-up of 7.4 years, there was no difference in the primary outcome between the two groups. In exploratory analyses, there were significantly fewer deaths from vascular events in the marine n-3 fatty acid arm (rate ratio: 0.81; 95% CI: 0.67–0.99), as well as a trend towards reduced risk of death from CHD (rate ratio: 0.79; 95% CI: 0.61–1.02). The Vitamin D and Omega-3 Trial (VITAL) trial randomised 25,871 healthy participants aged over 50 years (men) and 55 years (women) to receive marine n-3 fatty acids (840 mg/d EPA+DHA) and/or vitamin D (2000 IU/d) or placebo [54]. After a median follow-up of 5.3 years, there was no difference in the primary outcome of major cardiovascular events (a composite of MI, stroke or death from cardiovascular causes) in those participants supplemented with marine n-3 fatty acids versus placebo. An analysis of the individual components of the composite showed a significant reduction in the n-3 fatty acid arm in MI (hazard ratio: 0.72; 95% CI: 0.59–0.90) and CHD (hazard ratio: 0.83; 95% CI: 0.71–0.97). Correspondingly, there was also a reduced risk of death from these two non-prespecified outcomes (for MI—hazard ratio: 0.50, 95% CI: 0.26–0.97; for CHD—hazard ratio: 0.76, 95% CI: 0.49–1.16). Thus, whilst RCT evidence in primary prevention is less clear than that from the prospective cohort studies, there is now some indication of benefit from marine n-3 fatty acids towards cardiovascular health, especially CHD, from recent large and long RCTs, such as ASCEND and VITAL.

## 5. RCTs of Secondary Prevention of Cardiovascular Disease with Marine n-3 Fatty Acids

A number of large, randomized, controlled, secondary prevention trials or trials in high-risk patients have been conducted to investigate the effect of EPA and DHA in patients with established CVD. These trials generate a changing picture with time.

### 5.1. Secondary Prevention Trials and Meta-Analyses Published Prior to 2010

Several large, secondary prevention trials of marine n-3 fatty acids were conducted prior to 2010. The Diet and Reinfarction Trial (DART) included 2,033 recent (mean: 41 days) MI survivors, who were given dietary advice concerning fat, fish and fibre intake and followed up for 2 years [55]. Those patients advised to eat at least two portions of fatty fish per week (or to take fish oil supplements) had a 29% reduction in total mortality as well as a reduced risk of death from ischaemic heart disease at 2 years compared to those patients given other advice. The landmark Gruppo Italiano per lo Studio della Sopravvivenza nell’Infarto miocardico (GISSI)–Prevenzione trial investigated the effect of supplementation with 840 mg/d EPA+DHA in 11,324 recent (≤3 months) MI survivors versus vitamin E supplementation, supplementation with both EPA + DHA and vitamin E, and placebo control [56]. After 3.5 years, patients who received EPA + DHA had a 20% reduction in total mortality, 30% reduction in cardiovascular death and 45% decrease in sudden death compared to those that did not receive EPA + DHA. There was no benefit reported on non-fatal MI or stroke. The beneficial effect of EPA + DHA on total mortality and sudden cardiac death was observed after 3 months and 4 months of supplementation, respectively, and raised interest in the potential anti-arrhythmic action of EPA and DHA [57]. The GISSI investigators undertook a separate RCT in 6,975 patients with chronic heart failure (GISSI-HF) to investigate the effect of 840 mg/d EPA+DHA versus placebo over a period of ~4 years in this patient population [58]. The investigators reported a small (9%), but significant, reduction in all-cause mortality and a small (8%) reduction in combined all-cause mortality or admission to hospital for cardiovascular reasons in this high-risk population following supplementation with marine n-3 fatty acids. In line with the increased prescription of statins to prevent all-cause mortality and cardiovascular events at this time, JELIS directly investigated the use of 1.8 g/d EPA (as an ethyl ester) plus statin versus statin alone in 18,645 hypercholesterolaemic participants who were followed up for ~5 years [52]. As mentioned above, the addition of EPA to statin had no effect over statin alone in the primary prevention arm of JELIS, but in the secondary prevention arm, EPA caused a 19% decrease in non-fatal coronary events compared with the statin alone group [52]. Unlike GISSI, JELIS found no beneficial effect on cardiovascular mortality. A subsequent analysis found an inverse association between plasma EPA levels and risk of major coronary events; participants with the highest levels of plasma EPA (≥150 µg/mL) were 20% less likely to experience a major coronary event [59].

Meta-analyses of RCTs conducted prior to 2010 confirmed the reductions in mortality seen in individual trials of marine n-3 fatty acids ([60,61,62,63]; Table 3). For example, a 2002 meta-analysis of 11 RCTs in 15,806 patients with CHD found 20% lower risk of non-fatal MI, 30% lower risk of fatal MI, 30% lower risk for sudden death and 20% lower risk for all-cause mortality in patients who received marine n-3 fatty acids versus control [60]. Another meta-analysis from 2002 of 14 RCTs and 20,260 participants, both with and without CVD, found a 23% reduction in overall mortality and 32% reduction in cardiac mortality for those patients who were given marine n-3 fatty acids versus controls [61]. A further meta-analysis in 2009 focused on 8 RCTs and 20,997 patients with CHD found a 57% reduction in sudden death in patients with prior MI who were taking marine n-3 fatty acids compared with placebo [62]. In 2009, a meta-analysis of 11 RCTs representing 39,044 patients with all stages of CVD, including both low- and high-risk patients, found a 13% reduction in cardiovascular death and in sudden death and an 8% reduction in all-cause mortality in high-risk patients who were taking marine n-3 fatty acids compared to controls [63]. The investigators also found an 8% reduction in non-fatal cardiovascular events in moderate-risk patients.

### 5.2. RCTs with Marine n-3 Fatty Acids in High-Risk Patients Published in the Period 2010–2013

Three RCTs published in 2010 failed to replicate the findings of earlier trials [64,65,66]. The OMEGA study investigated the effect of supplementation with 840 mg/d EPA + DHA for 1 year in 3,851 early post-MI patients, with the primary endpoint of sudden cardiac death [64]. Marine n-3 fatty acids did not decrease the rate of sudden cardiac death, total mortality, major adverse cerebrovascular and cardiovascular events or revascularisation compared to control. The Supplémentation en Folates et Omega-3 (SU.FOL.OM3) trial investigated the effect of supplementation of B vitamins and/or marine n-3 fatty acids (600 mg/d EPA + DHA) in 2,501 patients with documented MI, unstable angina or ischaemic stroke for ~5 years [65]; the primary outcomes were cardiovascular death, stroke and non-fatal MI. Marine n-3 fatty acids did not have any effect on these outcomes. The Alpha Omega study included 4,837 post-MI patients who were given margarine fortified with low doses of EPA + DHA (400 mg/d), ALA (2 g/d), EPA + DHA + ALA or placebo, and followed up for 40 months [66]. There was no reduction in cardiovascular events in any group. On further analysis, those patients with diabetes in the EPA + DHA as well as ALA-fortified margarine groups, however, experienced a reduction in fatal CHD and arrhythmia-related events [66].

The Outcome Reduction with an Initial Glargine Intervention (ORIGIN) trial, published in 2012, assessed the effect of supplementation with 840 mg/d EPA + DHA in 12,536 dysglycaemic patients at high risk of cardiovascular events, together with insulin glargine or standard care for a median of 6 years [67]. No effect of marine n-3 fatty acids was reported for total mortality, death from cardiovascular causes or arrhythmia compared to placebo. The Risk and Prevention Study, published in 2013, investigated the effect of supplementation with 840 mg/d marine n-3 fatty acids in 12,513 patients with multiple cardiovascular risk factors or atherosclerotic vascular disease (but no MI) for a median of 5 years, and reported no effect on hospitalisation or death from cardiovascular causes compared to placebo [68]. In a prespecified subgroup analysis, compared with placebo, marine n-3 fatty acids resulted in an 18% lower incidence of the revised primary endpoint among women. Most of the secondary endpoints (e.g., fatal or non-fatal MI or stroke, fatal or non-fatal coronary event, and sudden death) did not differ between groups; however, admissions for heart failure were significantly lower in the marine n-3 fatty acid group.

Whilst these RCTs failed to show any benefit of marine n-3 fatty acids, they did have acknowledged limitations [69,70], which are worthy of mention. OMEGA was underpowered to detect any benefit on its primary outcome, sudden cardiac death, as power calculations were based on earlier RCTs in patients without more recent treatments and hence with higher background rates of sudden cardiac death compared to those seen in OMEGA. Furthermore, reported fish consumption was relatively high among the enrolled patients and increased during the trial, thereby increasing dietary EPA + DHA intake and introducing a confounding factor. The trial also had a relatively short follow-up period of just one year. Regarding SU.FOL.OM3, the length of time between the occurrence of primary cardiovascular event and the commencement of supplementation (median of 101 days) was longer than that of GISSI-Prevenzione (≤3 months) and the dose of EPA + DHA used was lower than that of GISSI-Prevenzione (840 mg/d EPA + DHA) and JELIS (1.8 g/d EPA). There were also fewer than expected background cardiac deaths, perhaps also due to more effective pharmacological management of cardiovascular risk factors. With respect to Alpha Omega, despite the long follow-up time, the dose of EPA+DHA used was modest and enrolment to the trial occurred on average at 4 years post-MI, despite the existence of data suggesting that early intervention with EPA + DHA is required to see a beneficial effect post-MI. ORIGIN included a large number of participants taking effective cardiovascular risk-reducing medications compared to earlier trials, and this may have made the cardioprotective effect of EPA + DHA harder to detect. The Risk and Prevention Study set out to examine the effect of marine n-3 fatty acids on the composite primary outcome of death, non-fatal MI and non-fatal stroke; however, the event rate at one year was lower than anticipated and the primary outcome was revised to hospitalisation or death from cardiovascular causes over the follow-up period.

Unsurprisingly, several meta-analyses conducted over the last ten years reflect the inclusion of these null RCTs and report more mixed conclusions than earlier meta-analyses ([21,22,71,72,73,74,75,76,77,78,79]; Table 3). A meta-analysis from 2013 (representing 11 RCTs and 15,348 patients with CVD) reported a 32% reduction in cardiac death, 33% reduction in sudden death and 25% reduction in myocardial infarction, with no effect on all-cause mortality or stroke [75]. This meta-analysis included only those RCTs with EPA + DHA dosages of at least 1 g/d and with a duration of at least 1 year. Another meta-analysis published in 2014, which included a broader range of marine n-3 fatty acid dosages and durations and focused only on patients with ischaemic CHD, reported that whilst there were no effects on cardiovascular events, there was a 12% reduction in death from cardiac causes, 14% reduction in sudden cardiac death and 8% reduction in all-cause mortality in those taking EPA + DHA versus placebo [76]. Published in 2017, a meta-analysis focusing solely on cardiac death as the outcome examined 14 RCTs (involving 71,899 subjects in both mixed and secondary prevention settings) with marine n-3 fatty acid supplementation for a duration of at least 6 months [77]. The study found an 8% lower risk of cardiac death in the primary analysis and a 13%–29% lower risk of cardiac death in those participants with intakes of EPA + DHA of at least 1g/d and for certain subgroups, such as subjects with high plasma triglycerides or cholesterol, and where <40% subjects were taking statins.

### 5.3. An Important RCT and a New Meta-Analysis were Published in 2019

**Figure 2:**
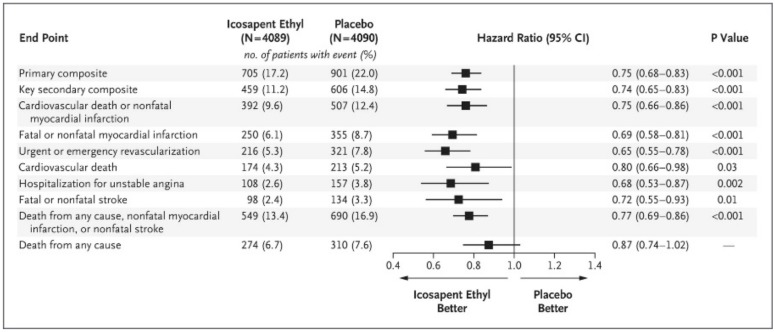
Effect of icosapent ethyl compared with placebo on different endpoints in REDUCE-IT [80]. “From New England Journal of Medicine, Bhatt D.L., Steg P.G., Miller M., Brinton E.A., Jacobson T.A., Ketchum S.B., Doyle R.T. Jr., Juliano R.A., Jiao L., Granowitz C., Tardif J.C., Ballantyne C.M.; REDUCE-IT Investigators, Cardiovascular Risk Reduction with Icosapent Ethyl for Hypertriglyceridemia, Volume No. 380, Page 11–22, Copyright © (2020) Massachusetts Medical Society. Reprinted with permission from Massachusetts Medical Society.”

The Reduction of Cardiovascular Events with Icosapent Ethyl–Intervention Trial (REDUCE-IT), published in early 2019, included 8,179 participants (29% in a primary prevention cohort with diabetes plus another cardiovascular risk factor and 71% in a secondary prevention cohort) supplemented daily with 4 g of icosapent ethyl (a highly purified EPA ethyl ester) or mineral oil placebo and followed up for a median of 4.9 years [80]. All patients were being treated with statins and had triglyceride concentrations of 135–499 mg/dL (1.52–5.63 mmol/L). The primary outcome was a composite of cardiovascular death, non-fatal MI, non-fatal stroke, coronary revascularisation or unstable angina. The patients who received icosapent ethyl had a statistically significant reduction in the primary outcome compared to placebo (hazard ratio: 0.75; 95% CI: 0.68–0.83; *p* < 0.001), the pre-specified secondary outcome (composite of cardiovascular death, non-fatal MI or non-fatal stroke) (hazard ratio: 0.80; 95% CI: 0.66–0.98; *p* = 0.03) and a whole range of other pre-specified outcomes (Figure 2). This effect was greater in the secondary prevention cohort than in the primary prevention cohort [80]. Interestingly, as with JELIS, REDUCE-IT used EPA only, but at a much higher dose (4 g daily versus 1.8 g daily) and included participants with high triglyceride levels and on statin medication. Accordingly, the participants in the icosapent ethyl arm had a significant reduction in triglycerides (a decrease of 0.5 mmol/L; *p* < 0.001) and LDL-cholesterol (a decrease of 0.13 mmol/L; *p* < 0.001) relative to placebo at 1 year. Of note, the improvement in the primary outcome in the icosapent ethyl arm of the trial was not dependent on the baseline triglyceride level or the degree of subsequent triglyceride lowering, suggesting that the reduction in cardiovascular risk in this population may be via a different mechanism independent of (or in addition to) triglyceride lowering. REDUCE-IT is of key interest as it demonstrates that even in at-risk populations that are well managed with modern pharmacological treatments, a suitably high dosage of EPA (i.e., 4 g daily) can provide an additional benefit in reducing cardiovascular-related events and mortality.

A recent meta-analysis, published in 2019, included data from 13 RCTs, including GISSI-Prevenzione, JELIS, GISSI-HF, SU.FOL.OM3, Alpha Omega, OMEGA, ORIGIN, VITAL, ASCEND and REDUCE-IT [81]. Trials had to have a sample size of at least 500 patients and a follow-up for at least one year to be included. The sample sizes ranged from 563 [82] to 25,871 [54], the mean duration of follow-up ranged from 1.0 [64] to 7.4 [53] years and the mean dose of EPA+DHA ranged from 0.37 [66] to 4.0 [80] g/d. The total sample size of the aggregated trials was 127,477, while the mean duration of follow-up was 5 years. The outcomes of interest included MI, CHD death, total CHD, total stroke, CVD death, total CVD and major vascular events. In the analysis excluding REDUCE-IT, marine n-3 fatty acid supplementation was associated with a significantly lower risk of MI, CHD death, CVD death and total CVD (Table 4). Inverse associations for all outcomes were strengthened after including REDUCE-IT (Table 4). Statistically significant linear dose–response relationships were found for several outcomes: for example, every 1 g/d EPA + DHA corresponded to 9% and 7% lower risk of MI and total CHD, respectively. The meta-analysis concluded that “marine [n-3 fatty acid] supplementation lowers risk for MI, CHD death, total CHD, CVD death, and total CVD, even after exclusion of REDUCE-IT. Risk reductions appeared to be linearly related to marine [n-3 fatty acid] dose.”

## 6. Trusted Authority Views on Marine n-3 Fatty Acids and Cardiovascular Disease

In 2010, the European Food Safety Authority (EFSA) concluded that EPA and DHA help to maintain normal cardiac function, normal blood pressure and normal blood concentrations of triglycerides in the general population [9]. In terms of the intake of EPA and DHA required to bring about these health claims, EFSA recommend an intake of 250 mg/day of EPA+DHA to maintain normal cardiac function, 3 g/d to maintain normal blood pressure and 2 g/d to maintain normal blood concentrations of triglycerides, as part of a healthy diet [9]. The American Heart Association (AHA) also supports the use of marine n-3 fatty acids. In 2018, the AHA published guidance in support of the dietary intake of fish in primary prevention [83]; specifically, the AHA advisory recommends 1 to 2 seafood meals per week to reduce the risk of congestive heart failure, CHD, ischemic stroke and sudden cardiac death. In recognition of the triglyceride-lowering effect of EPA and DHA, the AHA recently updated its earlier recommendation for the use of 2 to 4 g/d EPA + DHA for this indication: “we conclude that prescription n-3 fatty acids, whether EPA + DHA or EPA-only, at a dose of 4 g/d, are clinically useful for reducing triglycerides, after any underlying causes are addressed and diet and lifestyle strategies are implemented, either as monotherapy or as an adjunct to other triglyceride-lowering therapies” [84]. On the basis of the positive outcomes of REDUCE-IT, the European Society of Cardiology and the European Atherosclerosis Society issued an update to the “Clinical Practice Guidelines for the Management of Dyslipidaemias” specifically recommending that “in high-risk patients with [triglyceride] levels between 1.5 and 5.6 mmol/L (135–499 mg/dL) despite statin treatment, n-3 polyunsaturated fatty acids (icosapent ethyl 2 × 2 g/day) should be considered with a statin” [85]. Furthermore, the American Diabetes Association makes a recommendation “that icosapent ethyl be considered for patients with diabetes and atherosclerotic cardiovascular disease or other cardiac risk factors on a statin with controlled low-density cholesterol, but with elevated triglycerides (135–499 mg/dL) to reduce cardiovascular risk” [86]. Finally, the National Lipid Association’s position is “that for patients aged ≥45 years with clinical [atherosclerotic cardiovascular disease] ASCVD, or aged ≥50 years with diabetes mellitus requiring medication plus ≥1 additional risk factor, with fasting triglycerides 135 to 499 mg/dL on high-intensity or maximally tolerated statin therapy (±ezetimibe), treatment with icosapent ethyl is recommended for ASCVD risk reduction” [87]. In 2017, the AHA reinforced its earlier support for EPA+DHA in people with CVD [88] and extended [89] that “the recommendation for patients with prevalent CHD such as a recent MI remains essentially unchanged: Treatment with n-3 fatty acid supplements is reasonable for these patients. Even a potential modest reduction in CHD mortality (10%) in this clinical population would justify treatment with a relatively safe therapy. We now recommend treatment for patients with prevalent heart failure without preserved left ventricular function to reduce mortality and hospitalizations (9%) on the basis of a single, large RCT. Although we do not recommend treatment for patients with diabetes mellitus and prediabetes to prevent CHD, there was a lack of consensus on the recommendation for patients at high CVD risk. Because there are no reported RCTs related to the primary prevention of CHD, heart failure, and atrial fibrillation, we were not able to make recommendations for these indications.” In contrast to these well thought out and carefully worded statements by the AHA, in 2014, the National Institute for Health and Clinical Excellence in the UK recommended not to use marine n-3 fatty acids for the primary prevention of CVD, for the secondary prevention of CVD or in people with diabetes [90].

## 7. Summary, Discussion and Conclusions

There is a large body of evidence gathered from long-term prospective cohort studies that consistently demonstrates an association between higher intakes of fish, fatty fish and marine n-3 fatty acids (EPA+DHA) or higher levels of EPA and DHA in the body and lower risk of developing CVD, especially CHD, having an MI and cardiovascular mortality in the general population. This cardioprotective effect of EPA and DHA is plausible considering the robust identification of mechanisms that show that EPA and DHA beneficially modulate a number of known risk factors for CVD, such as blood lipids, blood pressure, heart rate and heart rate variability, platelet aggregation, endothelial function and inflammation. Despite this, evidence for primary prevention of CVD through RCTs is relatively weak. In high-risk patients, especially in the secondary prevention setting (e.g., post-MI), a number of large RCTs support the use of EPA + DHA (or EPA alone) as confirmed through a very recent meta-analysis [81]. Surveying these trials serves to highlight a number of factors which may have contributed to the positive outcomes reported and why other trials have had less conclusive or even null findings. Such factors include a sufficiently high dose of EPA alone or of a combination of EPA + DHA; sufficient duration of supplementation; supplementation in post-MI patients to begin relatively soon post-MI; and the adequate powering of studies to detect an effect on the primary outcome which may have a low rate of occurrence. Given these considerations, it is unsurprising that studies of short duration or using low doses of EPA + DHA conducted in the setting of multiple pharmaceutical (and other) approaches to controlling risk have failed to demonstrate the effects of EPA + DHA. As discussed elsewhere [91,92], there are also other factors to consider when evaluating and interpreting the literature in this field. The first is that the marine n-3 fatty acid naive condition is unlikely to occur, such that any placebo-controlled trial of EPA and DHA is conducted against a certain background intake of those fatty acids in all participants, although that background intake may be very low [5,6]. Nevertheless, background intakes of EPA and DHA can be highly variable both within and between individuals, with significant changes occurring simply by eating a single meal of fatty fish. Finally, the bioavailability of EPA and DHA can vary [93] (a) among individuals due to physiological differences, (b) according to the intake of meals in relation to supplements and (c) perhaps according to the time of day, thus influencing how much of these bioactive fatty acids is actually available to exert their effects. Taken together, these factors highlight the challenges to conducting robust trials in humans with supplemental n-3 fatty acids, and they are likely to contribute to the variable outcomes from such trials, especially when low doses of EPA and DHA are used.

Recent AHA advisories support the use of marine n-3 fatty acids in the treatment of hypertriglyceridemia [84] and in a range of patients with CVD [89] and the use of fish for primary prevention of CVD [83]. Both EPA and DHA beneficially modify a range of risk factors, although DHA may be more effective [37]. Nevertheless, the highly successful REDUCE-IT used pure EPA, although at a high dose [80]. Differentiating the dose-dependent effects of EPA and DHA on cardiovascular risk factors and on clinical outcomes is going to be important. Furthermore, robust primary prevention trials are still needed. In the meantime, recent trials reviewed herein and the most recent meta-analysis support the use of marine n-3 fatty acids to reduce cardiovascular mortality.

## Figures and Tables

**Table 1:** Content of EPA and DHA in fatty fish, lean fish, supplements and pharmaceuticals.

Fish Type	Typical EPA + DHA per Adult Serving	Comment
Fatty (e.g., salmon, trout, mackerel, sardines and herring)	1–3.5 g	Usually more EPA than DHA; content depends on the type of fish, season, water temperature, diet, stage of life cycle, wild or farmed and method of cooking
Lean (e.g., cod, plaice, haddock and sea bass)	0.1–0.3 g	Usually more EPA than DHA
**Supplement Type**	**Typical EPA + DHA Content per g of oil**	
Cod liver oil	200 mg	Usually more EPA than DHA
Standard “fish oil”	300 mg	Usually more EPA than DHA
Fish oil concentrate	450–600 mg	Usually more EPA than DHA
Tuna oil	460 mg	More DHA than EPA
Krill oil	205 mg	Usually more EPA than DHA; some in phospholipid form
Algal oil	400 mg	Mainly DHA
Flaxseed oil	0 mg	Contains α-linolenic acid, but not EPA or DHA
**Pharmaceuticals**	**Typical EPA + DHA Content per g of oil**	
Omacor/Lovaza	460 mg EPA + 380 mg DHA	In ethyl ester form
Omtryg	465 mg EPA + 375 mg DHA	In ethyl ester form
Epanova	550 mg EPA + 200 mg DHA	In free fatty acid form
Vascepa/icosapent ethyl	900 mg EPA	In ethyl ester form

Abbreviations: DHA, docosahexaenoic acid; EPA, eicosapentaenoic acid.

**Table 2:** Selected meta-analyses of the effect of marine n-3 fatty acids on cardiovascular risk factors.

Study	Cardiovascular Risk Factors Assessed	Study Design	Form and Dosage of n-3 Fatty Acids	Duration of n-3 Fatty Acid Treatment	Pooled Effects of n-3 Fatty Acids Versus Placebo
AbuMweis et al., 2018 [26]	Blood lipids, heart rate, blood pressure, inflammatory markers, platelet function and flow-mediated dilatation	Meta-analysis of 171 RCTs (up to Feb 2013) in participants in various states of health (note: the number of studies used for the analysis of different outcomes varied from 110 for triglycerides and HDL-cholesterol to 9 for flow-mediated dilatation)	Oral marine n-3 fatty acid supplements providing 0.18–15 g/d EPA+DHA	4–240 weeks	Significant dose-dependent decrease in triglycerides
					(MD = −0.368 mmol/L; 95% CI: −0.427–−0.309)
					Significant decrease in systolic blood pressure
					(MD = −2.195 mmHg; 95% CI: −3.171–−1.217)
					Significant decrease in diastolic blood pressure
					(MD = −1.37 mmHg; 95% CI: −2.415–−0.325)
					Significant decrease in heart rate (MD = −1.37 bpm; 95% CI: −2.41–−0.325)
					Significant decrease in CRP (MD = −0.343 mg/L; 95% CI: −0.454–−0.232)
					Significant increase in LDL-cholesterol (MD = 0.150 mmol/L; 95% CI: 0.058–0.243) and HDL-cholesterol (MD = 0.039 mmol/L; 95% CI: 0.024–0.054)
					No significant effect on total cholesterol, TNF-α, fibrinogen, platelet count, soluble intercellular adhesion molecule 1, soluble vascular cell adhesion molecule 1 or flow-mediated dilatation
Gao et al., 2013 [31]	Platelet aggregation	Meta-analysis of 15 RCTs (up to Jul 2011) including 742 participants in various states of health	Oral marine n-3 fatty acid supplements providing 0.84–6.8 g/d EPA+DHA	2–16 weeks	Significant decrease in adenosine diphosphate-induced platelet aggregation (SMD = −1.23; 95% CI: −2.24–−0.23; *p* = 0.02)
					Significant decrease in platelet aggregation units (SMD = −6.78; 95% CI: −12.58–−0.98; *p* = 0.02)
					Non-significant trend towards decreased collagen-induced and arachidonic acid-induced platelet aggregation
					Greater effect observed in non-healthy participants
Hidayat et al., 2017 [30]	Heart rate	Meta-analysis of 51 RCTs (up to May 2017) including ~3000 participants in various states of health	Oral marine n-3 fatty acid supplements providing 0.5–15.0 g/d EPA+DHA	2–52 weeks	Significant decrease in heart rate (WMD = −2.23 bpm; 95% CI: −3.07–−1.40); observed to be due to DHA, not EPA
Jiang et al., 2016 [36]	Pro-inflammatory eicosanoids	Meta-analysis of 18 RCTS (up to November 2015) including 826 subjects in various states of health	Oral marine n-3 fatty acid supplements providing 0.18–4.05 g/d EPA+DHA or EPA alone	4–24 weeks	Significant decrease in serum/plasma thromboxane B_2_ in participants with high risk of CVD (SMD = −1.26; 95% CI: −1.65–−0.86)
					Significant decrease in neutrophil leukotriene B_4_ in unhealthy subjects (SMD = −0.59; 95% CI: −1.02–−0.16)
Li et al., 2014 [35]	Pro-inflammatory cytokines	Meta-analysis of 68 RCTs (up to 2013) including 4601 participants in various states of health	Oral marine n-3 fatty acid supplements or dietary intake providing 0.3–6.6 g/d EPA+DHA	4–12 months	Participants with chronic disease:
					Significant decrease in CRP (WMD = −0.20 mg/L; 95% CI: −0.28–−0.12) and IL-6 (WMD = −0.22 pg/mL; 95%CI: −0.38–−0.06)
					No significant effect on TNF-α
					Healthy participants: Significant decrease in CRP (WMD = −0.18 mg/L; 95% CI: −0.28–−0.08) and TNF-α (WMD = −0.12 pg/mL; 95% CI: −0.16–−0.07)
					No significant effect on IL-6
Miller et al., 2014 [27]	Blood pressure	Meta-analysis of 70 RCTs (up to February 2013) in normotensive and hypertensive subjects	Oral marine n-3 fatty acids from seafood, fortified foods, fish oil, algal oil and purified ethyl esters; mean EPA+DHA dose: 3.8 g/d	>3 weeks (mean study duration: 69 days)	Significant decrease in systolic blood pressure (WMD = −1.52 mmHg; 95% CI: −2.25–−0.79)
					Significant decrease in diastolic blood pressure (WMD = −0.99 mmHg; 95% CI: −1.54–−0.44)
					Significant decrease in systolic blood pressure (WMD = −4.51 mmHg; 95% CI: −6.12–−2.83) and diastolic blood pressure (WMD = −3.05 mmHg; 95% CI: −4.35–−1.74) in hypertensive individuals
Mozaffarian et al., 2005 [28]	Heart rate	Meta-analysis of 30 RCTs (up to January 2005) including 1678 healthy participants	Oral marine n-3 fatty acid supplements; median EPA+DHA intake: 3.5 g/d	>2 weeks (median study duration: 8 weeks)	Significant decrease in heart rate (WMD = −1.6 bpm; 95% CI: 0.6–2.5)
					In those with baseline heart rate ≥ 69 bpm, heart rate decreased by 2.5 bpm (95% CI: 1.4–3.5)
Pase et al., 2011 [34]	Arterial stiffness	Meta-analysis of 10 RCTs (up to September 2010) including 550 participants in various states of health	Oral marine n-3 fatty acid supplements providing 0.64–3 g/d EPA+DHA	6–105 weeks	Significant improvement in pulse wave velocity (SMD = 0.33; 95% CI: 0.12–0.56)
					Significant improvement in arterial compliance (SMD = 0.48; 95% CI: 0.24–0.72)
Wang et al., 2012 [33]	Vascular endothelial function	Meta-analysis of 16 RCTs (up to August 2011) including 901 participants in various states of health	Oral marine n-3 fatty acid supplements and dietary intake providing 0.45–4.7 g/d EPA+DHA	2 weeks to 12 months (median: 56 days)	Significant increase in flow-mediated dilatation (WMD = 2.3%; 95% CI: 0.89–3.72)
					No significant change in endothelium-independent vasodilation
Xin et al., 2012 [32]	Vascular endothelial function	Meta-analysis of 16 RCTs (up to February 2012) including 1385 participants in various states of health	Oral marine n-3 fatty acid supplements providing 0.45–4.53 g/d EPA+DHA	2-52 weeks	Significant increase in flow-mediated dilatation (WMD = 1.49%; 95% CI: 0.48–2.5)
Xin et al., 2013 [29]	Heart rate variability	Meta-analysis of 15 RCTS including 692 participants in various states of health	Oral marine n-3 fatty acid supplements providing 0.64–5.9 g/d EPA+DHA	6-24 weeks	Significant increase in high frequency power value of heart rate variability (SMD = 0.30). A sensitivity analysis demonstrated a significant reduction in low frequency power/high frequency power ratio with >1 g/d EPA+DHA

Abbreviations: CI, confidence interval; CVD, cardiovascular disease; CRP, C-reactive protein; DHA, docosahexaenoic acid; EPA, eicosapentaenoic acid; HDL, high-density lipoprotein; IL-6, interleukin-6; LDL, low-density lipoprotein; MD, mean difference; RCT, randomized controlled trial; SMD, standard mean difference; TNF-α, tumour necrosis factor-α; WMD, weighted mean difference.

**Table 3:** Meta-analyses published up to 2018 of RCTs investigating the effect of marine n-3 fatty acids on cardiovascular outcomes.

Study	Study Design	Form & Dosage of Marine-3 Fatty Acids	Duration of Treatment with Marine n-3 Fatty Acids	Pooled Effects of Marine n-3 Fatty Acids Versus Placebo
Bucher et al., 2002 [60]	11 RCTs (up to August 1999) representing 15,806 patients with CHD	Dietary (2 RCTs) and supplemental (9 RCTs) marine n-3 fatty acids with a dose range of 0.3–6.0 g/d EPA and 0.6–3.7 g/d DHA	6–46 months (mean: 20 months)	30% reduction in fatal MI
				30% reduction in sudden death
				20% reduction in overall mortality
Studer et al., 2005 [61]	14 RCTs (up to June 2003) representing 20,260 participants in primary and secondary prevention settings	Supplemental marine n-3 fatty acids; dose range not given	Mean: 1.9 ± 1.2 years	23% reduction in overall mortality
				32% reduction in cardiovascular mortality
Zhao et al., 2009 [62]	8 RCTs (up to June 2008) representing 20,997 patients with CHD	Dietary (3 RCTs) and supplemental (5 RCTs) marine	9–108 months (mean: 33 months)	57% reduction in sudden death in patients with prior MI
				39% increased risk of sudden death in patients with angina
		n-3 fatty acids with a dose range of 0.3–4.1 g/d EPA and 0.4–2.8 g/d DHA		29% reduction in cardiac death (NS)
				23% reduction in all-cause mortality (NS)
Marik and Varon, 2009 [63]	11 RCTs (up to December 2008) representing 39,044 patients with all stages of CVD including high-risk and low-risk subjects	Supplemental marine n-3 fatty acids with a dose range of 0.7–4.8 g/d EPA + DHA (mean: 1.8 ± 1.2 g/d)	1–4.6 years (mean: 2.2 ± 1.2 years)	13% reduction in cardiovascular death in high-risk patients
				13% reduction in sudden cardiac death in high-risk patients
				8% reduction in all-cause mortality in high-risk patients
				8% reduction in non-fatal cardiovascular events in moderate-risk patients.
Kotwal et al., 2012 [71]	20 RCTs (up to March 2011) representing 62,851 patients in primary and secondary prevention settings	Diet (3 RCTs) and supplemental (17 RCTs) marine n-3 fatty acids with a dose range of 0.8–3.4 g/d EPA + DHA	6 months–6 years	14% reduction in vascular death
				No effect on cardiovascular events, total mortality, coronary events, arrhythmia or cerebrovascular events
Kwak et al., 2012 [72]	14 RCTs (up to April 2011) representing 20,485 patients with CVD	Supplemental marine n-3 fatty acids with a dose range of 0.4–4.8 g/d EPA + DHA (mean: 1.7 g/d EPA + DHA)	1–4.7 years (mean: 2 years)	9% reduction in cardiovascular death
				No effect on cardiovascular events, all-cause mortality, sudden cardiac death, MI, congestive heart failure or stroke
Trikalinos et al., 2012 [73]	18 RCTs (up to May 2011) representing 51,264 patients	Supplemental marine n-3 fatty acids with a dose range of 0.27–6.0 g/d EPA + DHA	1–5 years	11% reduction in cardiovascular mortality
Rizos et al., 2012 [74]	20 RCTs (up to August 2012) representing 68,680 patients in primary and secondary prevention settings	Diet (2 RCTs) and supplemental (18 RCTs) marine n-3 fatty acids with a dose range of 0.53–1.80 g/d EPA + DHA (median EPA + DHA dose: 1 g/d)	1–6.2 years (median: 2 years)	No effect on all-cause mortality, cardiac death, sudden death, MI or stroke
Casula et al., 2013 [75]	11 RCTs (up to March 2013) representing 15,348 patients with CVD	Supplemental marine n-3 fatty acids with a dose range of 1–6 g/d EPA + DHA	≥ 1 year (duration ranged from 1–3.5 years)	32% reduction in cardiac death
				33% reduction in sudden death
				25% reduction in MI
				11% reduction in all-cause mortality (NS)
				No effect on stroke
Wen et al., 2014 [76]	14 RCTs (up to May 2013) representing 32,656 patients with CHD	Supplemental marine n-3 fatty acids with a dose range of 0.4–6.9 g/d EPA + DHA	< 3 months to 4.6 years	12% reduction in death from cardiac causes
				14% reduction in sudden cardiac death
				8% reduction in all-cause mortality
				7% reduction in cardiovascular events (NS)
Chowdhury et al., 2014 [21]	17 RCTs (up to June 2013) representing 76,580 participants	Supplemental marine n-3 fatty acids with a dose range of 0.3 g/d EPA to 6 g/d EPA + DHA.	0.1–8 years	7% reduction in coronary outcomes (NS)
Alexander et al., 2017 [22]	18 RCTs (up to November 2015)	Supplemental marine n-3 fatty acids with a dose range of 0.4–5.0 g/d EPA + DHA	0.5–7 years	14%–16% reduction in CHD in high-risk subgroups i.e., those with elevated triglycerides and LDL-cholesterol
Maki et al., 2017 [77]	14 RCTs (up to December 2016) representing 71,899 patients in a mixed/secondary prevention setting	Supplemental marine n-3 fatty acids with a dose range of 0.27–5.0 g/d EPA + DHA	≥ 6 months (range 0.5–6.2 years)	8% reduction in cardiac death
				~13%–29% reduction in cardiac death in the subgroup with high-risk individuals (secondary prevention, high triglycerides, high LDL-cholesterol and <40% statin use) and with EPA+DHA > 1 g/d
Aung et al., 2018 [78]	10 RCTs representing 77,917 high-risk patients (prior CHD or stroke)	Supplemental marine n-3 fatty acids with a dose range of 0.2–1.8 g/d EPA and 0–1.7 g/d DHA	1–6.2 years (mean: 4.4 years)	7% reduction in CHD death (NS)
				No effect on non-fatal MI, CHD events or major vascular events
Abdelhamid et al., 2018 [79]	79 RCTs (up to April 2017) representing 112,059 participants in primary and secondary prevention settings	Dietary or supplemental marine n-3 fatty acids with a dose range from 0.5 g/d to ~5 g/d EPA + DHA	1–7 years	7% reduction in CHD events
				No effect on all-cause mortality, cardiovascular mortality, cardiovascular events, CHD mortality, stroke or arrhythmia.

Abbreviations: CHD, coronary heart disease; DHA, docosahexaenoic acid; EPA, eicosapentaenoic acid; LDL, low-density lipoprotein; MI, myocardial infarction; NS, not significant; RCT, randomised controlled trial.

**Table 4:** Summary of findings from the meta-analysis published by Hu et al. [81] in 2019.

Outcome	Number of Studies	Finding for Marine n-3 Fatty Acids Versus Placebo	Finding if Data from REDUCE-IT Removed
		Rate Ratio [95% Confidence Interval] (*p*)	Rate Ratio [95% Confidence Interval] (*p*)
Myocardial infarction	13	0.88 [0.83, 0.94] (<0.001)	0.92 [0.86, 0.99] (0.020)
CHD death	12	0.92 [0.86, 0.98] (0.014)	Outcome not reported in REDUCE-IT
Total CHD	13	0.93 [0.89, 0.96] (<0.001)	0.95 [0.91, 0.99] (0.008)
Total stroke	13	1.02 [0.95, 1.10] (0.569)	1.05 [0.98, 1.14] (0.183)
CVD Death	12	0.92 [0.88, 0.97] (0.003)	0.93 [0.88, 0.99] (0.013)
Total CVD	13	0.95 [0.82, 0.98] (<0.001)	0.97 [0.94, 0.99] (0.015)
Major vascular events	13	0.95 [0.93, 0.98] (<0.001)	0.97 [0.94, 1.00] 90.058)

Abbreviations: CHD, coronary heart disease; CVD, cardiovascular disease.

## Abbreviations

AHA	American Heart Association
ALA	alpha-linolenic acid
CHD	coronary heart disease
CI	confidence interval
CRP	C-reactive protein
CVD	cardiovascular disease
DHA	docosahexaenoic acid
DPA	docosapentaenoic acid
EFSA	European Food Safety Authority
EPA	eicosapentaenoic acid
HDL	high-density lipoprotein
IL	interleukin
LDL	low-density lipoprotein
MD	mean difference
MI	myocardial infarction
NS	not significant
RCT	randomised controlled trial
SMD	standard mean difference
TNF	tumour necrosis factor
WMD	weighted mean difference
